# Barriers and benefits of mHealth for community health workers in integrated community case management of childhood diseases in Banda Parish, Kampala, Uganda: a cross-sectional study

**DOI:** 10.1186/s12875-024-02430-4

**Published:** 2024-05-20

**Authors:** Winnifred K. Kansiime, Edwinah Atusingwize, Rawlance Ndejjo, Emmanuel Balinda, Moses Ntanda, Richard K. Mugambe, David Musoke

**Affiliations:** 1 Department of Disease Control and Environmental Health, School of Public Health, College of Health Sciences, Makerere University, P.O. Box 7072, Kampala, Uganda; 2 Department of Networks, College of Computing and Information Science, Makerere University, P.O. Box 7072, Kampala, Uganda

**Keywords:** Village health team, Community health workers, mHealth, Barriers, Benefits, Informal settlement

## Abstract

**Background:**

Low-quality data presents a significant challenge for community health workers (CHWs) in low and middle-income countries (LMICs). Mobile health (mHealth) applications offer a solution by enabling CHWs to record and submit data electronically. However, the barriers and benefits of mHealth usage among CHWs in informal urban settlements remain poorly understood. This study sought to determine the barriers and benefits of mHealth among CHWs in Banda parish, Kampala.

**Methods:**

This qualitative study involved 12 key informant interviews (KIIs) among focal persons from Kampala City Council Authority (KCCA) and NGOs involved in data collected by CHWs, and officials from the Ministry of Health (MOH) and two mixed-sex Focused Group Discussions (FGDs) of CHWs from Banda parish, Kampala district. Data analysis utilised Atlas Ti Version 7.5.7. Thematic analysis was conducted, and themes were aligned with the social-ecological model.

**Results:**

Three themes of institutional and policy, community and interpersonal, and individual aligning to the Social ecological model highlighted the factors contributing to barriers and the benefits of mHealth among CHWs for iCCM. The key barriers to usability, acceptability and sustainability included high training costs, CHW demotivation, infrastructure limitations, data security concerns, community awareness deficits, and skill deficiencies. Conversely, mHealth offers benefits such as timely data submission, enhanced data quality, geo-mapping capabilities, improved CHW performance monitoring, community health surveillance, cost-effective reporting, and CHW empowering with technology.

**Conclusion:**

Despite limited mHealth experience, CHWs expressed enthusiasm for its potential. Implementation was viewed as a solution to multiple challenges, facilitating access to health information, efficient data reporting, and administrative processes, particularly in resource-constrained settings. Successful mHealth implementation requires addressing CHWs’ demotivation, ensuring reliable power and network connectivity, and enhancing capacity for digital data ethics and management. By overcoming these barriers, mHealth can significantly enhance healthcare delivery at the community level, leveraging technology to optimize resource utilization and improve health outcomes. mHealth holds promise for transforming CHW practices, yet its effective integration necessitates targeted interventions to address systemic challenges and ensure sustainable implementation in LMIC contexts.

**Supplementary Information:**

The online version contains supplementary material available at 10.1186/s12875-024-02430-4.

## Introduction

Globally, Community health workers (CHWs) are influential actors in frontline health services [1]. In the communities, they play a significant role in preventing and controlling diseases, particularly in Low or Middle-Income Countries (LMICs) [2–5]. Low-quality data are among the challenges CHWs face in LMICs [6, 7]. CHWs in Uganda, known as Village Health Teams (VHTs), mobilize communities for health services, promote health within communities at the household level, link and refer the population to healthcare facilities (HCFs) [8]. In Uganda, CHW roles include Integrated Community Case Management (iCCM) of childhood killer diseases, which includes assessment/diagnosis, treatment and referral for diarrhoea, pneumonia, and malaria in children under five years of age and registering all community-identified cases [9]. They collect data using VHT registers, which are transferred into the Healthcare facility (HCF) summary form during quarterly meetings and later linked to the health management information system (HMIS) data. The VHT/iCCM register is used to record the cases, other health indicators and basic demographics of the sick children as the CHWs interact with the community. However, CHWs face several challenges such as inadequate supervision, poor motivation and lack of incentives, poor retention, and limited training [10] on data collection often resulting in poor-quality data. The recording, sharing, and accessing of data records in VHT registers is a manual process which is prone to human errors during recording in addition to data manipulation. This undermines the reliability and validity of the data. Yet, this data informs HCFs, district hospitals, referral hospitals, the Ministry of Health (MoH), and associated non-governmental organisations (NGOs) in their decision-making processes. Additionally, the delay in submitting the reports misses an opportunity for early identification of outbreaks in communities. To ensure the quality of data, supervisors are in place to oversee the work done by CHWs which includes checking the quality of data collected. However, poor-quality data persists among CHWs in many parts of the country.

Mobile application systems for health (mHealth) allow CHWs to electronically record data and submit reports while limiting manual inputs [9]. In rural Uganda, mHealth has been shown to increase CHWs’ knowledge of signs and symptoms/danger signs of childhood illnesses through a health education intervention [11]. However, most mHealth studies among CHWs have focused on rural areas [12–14], leaving out urban informal settlements, which continue to grapple with different CHWs challenges.

There is limited knowledge of CHWs using mHealth in the informal settlements of Kampala to collect iCCM data. Additionally, the existing barriers and benefits to mHealth use by CHWs for iCCM in urban areas remain unknown. Knowledge of the barriers and benefits to the diffusion of mHealth innovation through usability, acceptability and sustainability by CHWs is critical to guide implementation, investment, and policies. Therefore, this study sought to determine the barriers and benefits to mHealth among CHWs for iCCM in Banda parish, Kampala, in order to inform policymakers and other stakeholders on the development of good practices and policies that could guide the integration of mHealth into iCCM-related health promotion models for primary care.

## Methods

### Study setting and population

The study was conducted in Kampala district, Uganda’s capital, in February 2022. Kampala has five divisions: Central, Rubaga, Nakawa, Makindye, and Kawempe. Informal settlement dwellers contribute 31% of Uganda’s urban population [15]. In Kampala alone, about 40% of the population dwells in informal settlements with limited basic infrastructure and the land profile location is near wetlands [16]. These settlements are characterised by congestion, crowding of housing structures, dilapidated and unregulated housing structures that generally have poor ventilation, inadequate water, sanitation and hygiene access, limited services and infrastructure, and low government response to needs and services [17] creating conditions for high morbidity and mortality rates of children under five years [18, 19]. The Kampala population profile constitutes 88% of youth aged 15–35 [20]. Nakawa division was selected for the study because it had the highest morbidity for children aged 0–4 years in Kampala, 31.5% [21].

Banda informal settlement was purposively selected in Nakawa division because of its proximity to HCFs in the area that would support the CHWs’ role of data collection. Banda is located on the eastern outskirts of Kampala, Uganda’s capital city. It has about 10,000 households with a household size of five and covers approximately 150 acres. It consists of mainly rural-urban immigrants of several ethnic groups. Most dwellers have low levels of education and some uneducated and engage in informal livelihoods. The study population included CHWs, CHW focal persons/supervisors, in-charges of HC IIIs and IVs, Division health officers, KCCA and MOH officials in charge of the health management information system (HMIS), and NGOs involved in data collected by CHWs.

### Study design and sampling

This was a qualitative formative study, part of a larger study that used the Human Centred design framework to assess the quality of integrated community case management data collected by CHWs using mHealth data collection tools. In the empathise phase of the larger study, this formative study determined the barriers and benefits to mHealth among CHWs for iCCM by using the Social-Ecological Model (SEM) to analyse the data. It employed 12 key informant interviews (KIIs) among focal persons from KCCA and NGOs involved in data collected by CHWs, and officials from the MOH and two mixed-sex Focused Group Discussions (FGDs) of CHWs from Banda parish. The KIs were recruited based on their knowledge and experience of working with CHWs, community-level health data, and mHealth. CHWs were included based on their involvement in iCCM. Through the division medical officer, the CHW coordinator and her assistant mobilised the CHWs and selected the venue for this activity. The study team and purpose was introduced to the CHWs by the CHW coordinator. These steps ensured a smooth community entry and led to trust between the CHWs and the study investigators throughout the study. FGDs with CHWs allowed for exploring opinions and experiences that would have been less forthcoming in a one-to-one interview [22].

### Data collection tools

KIIs were conducted in English using a key informant interview guide with questions on the perceptions, experiences, barriers and benefits of mHealth for CHWs, and the potential challenges of mHealth integration into iCCM. The FGDs were conducted using an FGD guide with similar questions translated into *Luganda*. The data collection tools were pretested with Key Informants and CHWs from an informal settlement that was not part of the study. Research assistants provided feedback about the effectiveness of the questions’ clarity instructions.' Necessary revisions were made.

### Data collection

The study investigators conducted a 2-day training of Research Assistants on data collection to ensure data quality. Appointments were set up with the KIs at their place of work or online using the Zoom platform. For FGDs, meetings were conducted at Banda community hall within the Parish. Interviews were conducted by the study investigators and trained research assistants with Public Health training and experience in conducting qualitative research. The FGDs included male and female participants, with one having 15 CHWs and the other 12 CHWs. Both FGDs and KIIs lasted between about an hour.

### Data management

Data were digitally recorded using audio recorders and zoom recording and transcribed into Microsoft Word documents for analysis by experienced data collectors. In the case of FGDs with CHWs, these were translated into English at the time of transcription. Transcripts were checked against the audio files. Experienced qualitative researchers conducted qualitative data analysis. Atlas Ti Version 7.5.7 was used to code transcripts and assist the analysis process. Analysis was conducted using a thematic framework approach. Data coding was applied by using a deductive approach to create themes and sub-themes as they emerged from the transcripts. The research team convened regularly to debate differences in coding and interpretation. Following coding, we used the Social-Ecological Model (SEM) [23] as an organizing framework to present the data. This ecological model recognises systems’ interaction at multiple levels, from individual to broader societal levels, influencing program acceptance. The model facilitated the exploration of dynamic interactions across different social levels. Emerging themes and subthemes that could impact the success and diffusion of an mHealth for iCCM intervention were grouped into three different SEM levels and presented accordingly in the results section, starting with the Institutional and policy level, interpersonal and community levels, and finally, the individual level. The preliminary findings were presented to the CHWs, KCCA, MOH officials, and other stakeholders to collaborate on the findings and finalize the narrative per theme.

### Ethical considerations

Ethical approval for the study was obtained from Makerere University School of Public Health Research Ethics Committee (SPH-2021-173) and Uganda National Council for Science and Technology (HS1983ES). Administrative clearance was sought from the Kampala City Council Authority (KCCA), which presides over the study area. Information sheets and consent forms were available in the local language (Luganda) or English with details on the purpose of the project, procedures to be followed, and the risks and benefits of participation. Written informed consent to participate in the study was sought from all study participants.

## Results

**Fig. 1:**
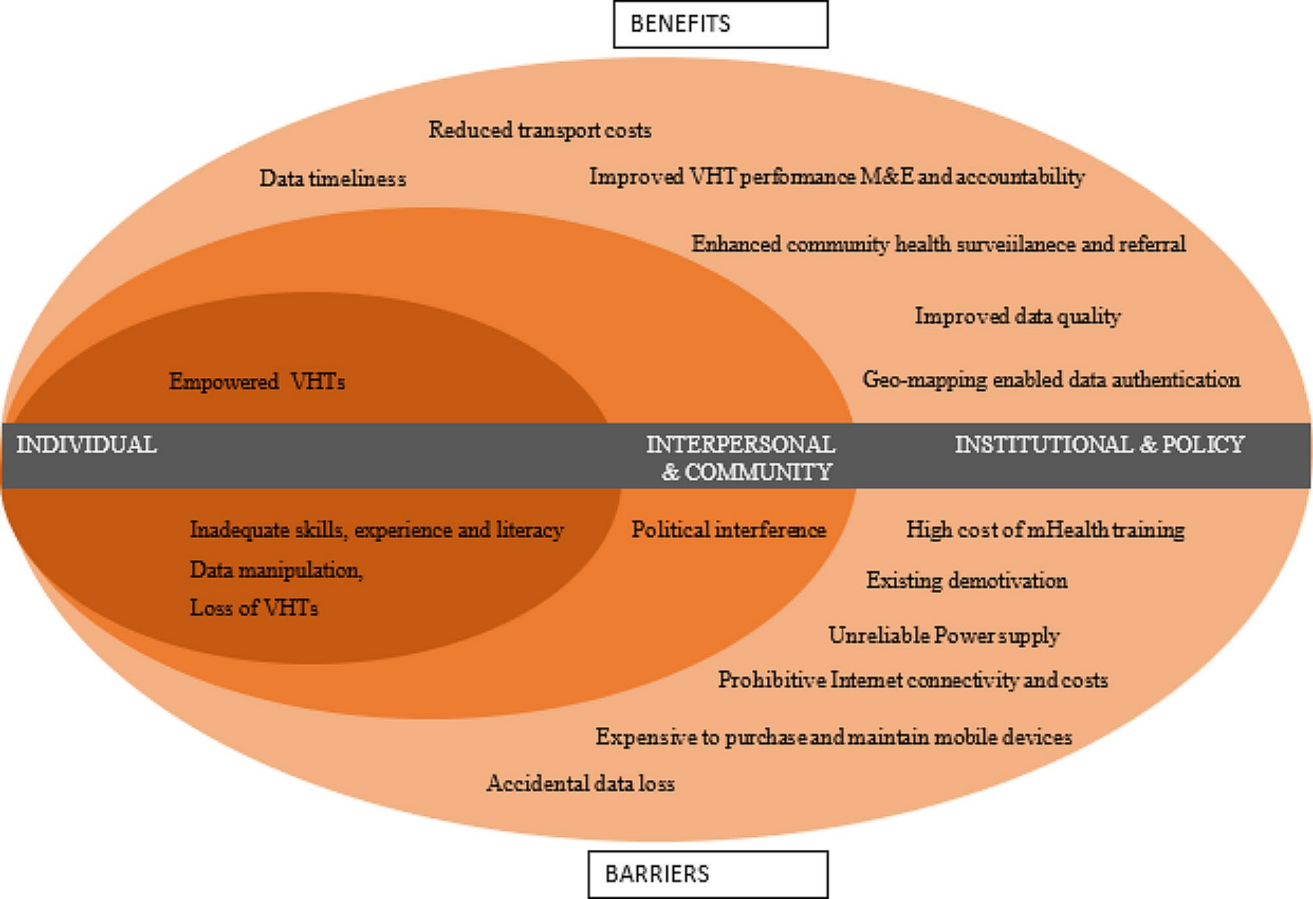
Social-ecological model with layers of barriers and opportunities for mHealth for village health teams iCCM data among CHWs

Three themes described the barriers and benefits to mHealth among CHWs. These were individual, interpersonal and community, and institutional and policy. The SEM model was used to organize these barriers and benefits. Under these, various sub-themes emerged that may be barriers or benefits affecting or arising from the mHealth among the CHWs in the community (Fig. 1).

### Barriers that may affect mHealth for CHWs for iCCM data

#### Institutional and policy barriers

Four subthemes revealed how institutional and policy barriers may affect the usability, acceptability and sustainability of mHealth among CHWs in Banda informal settlements. These were high costs and expenditures in training CHWs on mHealth, existing demotivation among CHWs, infrastructure challenges (unreliable power supply, prohibitive internet connectivity and costs, expensive mobile device purchase, and maintenance), and accidental data loss.

#### High costs and expenditures in training CHWs on mHealth

For the usability of mHealth to be effective, training of CHWs was a requirement. This would require frequent sessions to ensure all CHWs understand the digital tools and are continuously updated with the system. Frequent trainings for CHWs to use mobile phones for health data collection would require significant time and resources. However, there is limited capacity and funding to support such training initiatives. The challenge of training CHWs to use mobile phones for data collection becomes more pronounced when dealing with older CHWs.

*“Frequent trainings [for CHWs] will have to be organised which will necessitate a lot of time and materials to be distributed and yet there is no capacity to support those trainings with the CHWs, due to the fact that the service is voluntary, there are basically no funds to initiate such technology which will be very difficult.’ KI 7*.

*‘Because these CHWs are becoming old, trainings them to use mobile phones in health data collection becomes expensive, due to the fact that they will need continuous reminders all the time because they tend to forget, which is not possible due to limited resources within the community.’ FGD 1*.

#### Existing demotivation among CHWs

There were inadequate supplies and equipment needed for CHW activities. For instance, the current lack of proper identification materials, such as IDs and T-shirts faced by CHWs in several communities, resulted in the hesitance of community members to provide information to CHWs, which might broadly impact mHealth. Respondents emphasised that CHW work is a pro bono service performed without adequate facilitation. For instance, a refund for transport costs during meetings and reporting between health centres and different communities was often inadequate or lacking for some CHWs. Overall, the existing demotivation factors of CHWs may act as barriers to mHealth.

*‘There was an m-reporting program that was started up by an organization, which involved the use of phones as the main reporting tool for the CHWs in an urban area …nevertheless, there were some complaints which were raised by these CHWs, for instance, lack of enough facilitation, airtime and other problems…” KI 3*.

#### Infrastructure challenges

Most respondents were worried that, overall, the infrastructure and systems required to sustain the mhealth application were not available and are difficult to develop and sustain.

#### Unreliable power supply

Unreliable power supply/electricity would affect the charging of phones and yet the success of mHealth relied on having fully charged phones was a concern for all respondents. They indicated that power shortage and unreliability at individual and community level was expected and would consequently affect data quality, delay reports, and make data collection expensive and time-consuming for CHWs. Rainy seasons can exacerbate the challenges of charging phones in some communities. It may result in power outages and a shortage of electricity supply, making it even more challenging for CHWs to maintain phone battery levels.

*“The phones should always be charged which is quite difficult in some areas. Additionally, during rainy seasons, in some communities, there is a shortage of electricity, therefore if the VHT runs short of phone battery in the field during that season, data collection will cease at that moment, which not only makes submissions slow but also makes CHWs’ work very expensive because he/she will have to go back to that same community time and again to collect data.” FGD 1*.

#### Prohibitive internet connectivity and costs

Most respondents were concerned that some communities’ intermittent or unreliable network connectivity would affect data collection and submission processes. Another challenge was related to the inability to purchase and maintain the mobile data and airtime needed by CHWs sustainably to enhance data quality and capture using phones. Internet servers were cited as a huge investment by organizations implementing mHealth, which could affect the development and sustainability of mHealth.

*“mHealth would come along with issues of infrastructures, internet interruptions which might sometimes delay data submissions to the data server. The other challenge would be inadequate mobile data and airtime to enhance data quality and capture.” KI 2*.

#### Expensive to purchase and maintain mobile devices

Key informants generally felt that for electronic platforms to work efficiently, considerable investments are required to buy SMART mobile phones, which may be unaffordable for both CHWs nationally and partner organisations. There were concerns about phone misuse, leading to phone damage or loss, such as phone malfunctioning and mHealth app malfunctioning. If the app fails to work, it can hinder data collection, leading to delays in data submissions and potentially impacting the system’s efficiency.

*“…CHWs will need phones of good quality which is very costly and expensive.” KI 2*.

*‘Secondly, it is expensive to purchase those phones because it involves an extra cost …maintenance is also another issue because some of the CHWs misuse these phones which are provided to them, some may get lost, while others might stop functioning and many others. Some of the other challenges that we grapple with is that the app reaches a moment when it cannot work and if it fails to work, there will be no data collection, and therefore data submissions will be slowed down.” KI 10*.

#### Accidental data loss

Accidental data loss may result from (i) possible misuse of phones by some of the CHWs who may share them with other people and (ii) possible phone theft. The nature of the work of CHWs involves operating in communities sometimes until late in the evening, creating a possibility of phones being stolen, especially as some of the communities in the informal settlements tend to be unsafe.

### Interpersonal and community barriers

Under this theme, two subthemes were reported as the potential barrier to mHealth.

#### Trust and reluctance to share health information

Some community members may be hesitant to provide their health information if other members, particularly those selected to use mobile phones for data collection, are perceived as less trustworthy than the CHWs they know and trust. This can lead to reluctance to share sensitive health information with unfamiliar individuals, even if they are equipped with mHealth tools.

#### Limited sensitisation and awareness about mHealth in communities

The FGDs emphasized insufficient sensitisation or education about the primary goal and objectives of using mobile phones for data collection in communities. Without proper awareness, community members may not fully understand the purpose and benefits of the new data collection mechanism. Due to the lack of sensitisation, community members might display hostility and rigidity towards providing their health data information. They may be hesitant to participate in data collection using mobile phones as they are accustomed to traditional methods such as VHT registers. Community members might distrust the new data collection approach, fearing that their information might be used for other purposes without their consent. This mistrust can create bias, leading to incomplete or inaccurate data submissions, and it may result in community members being unresponsive to questions about their health.

*‘Because of limited or no sensitisation about the major goal and objective of data collection using mobile phones in communities…since people in the community are not sensitised, they will tend to be hostile and very rigid to provide their health data information, leading to doubts concerning the data submitted to health facilities.” FGD 1*.

“*People are used to seeing us using the logbooks, but as a mechanism of data collection is transformed to using mobile phones, and because they are not sensitised, they will tend to be rigid and disrespectful to us thinking that we might use their information for something else, creating bias or not responding to questions about their health.” FGD 1*.

### Individual barriers

Three subthemes revealed how individual factors may act as barriers to mHealth among CHWs. These included inadequate skills, experience, and literacy, data manipulation, and CHWs short-term commitment and involvement

#### Inadequate skills, experience, and literacy

Respondents were concerned that some CHWs lack enough experience and skills regarding mHealth, and most have low literacy levels. They were afraid that while mHealth use is promising, the elderly CHWs would be excluded from the process. For example, respondents were worried that the elderly CHWs could be eliminated as they would find using technology difficult. However, most of the elderly CHWs are the most experienced, trusted and dedicated, and eliminating them would negatively impact essential aspects of CHW, especially community mobilisation.

*“The barriers in using mHealth also depend on the literacy levels of the VHT…” KI 2*.

**“***The challenge is that most CHWs who have delegated all their time serving the people in their communities are becoming old whereby they take long to pick up with the use of new technology… more to this is that people in the community trust these CHWs, community members tend to be hesitant to provide their health information if other members are selected to collect health data using mobile phones in these communities.” FGD I*.

*“…, there are some elderly CHWs who are good at mobilization, but might not be good in using the technology, and adapting with the technology may tend to be very difficult and time-consuming for these elderly CHWs.” KI 2*.

#### Data manipulation

Data manipulation was another issue raised as a possible challenge to data quality arising from using mHealth. For instance, discussions indicated that because of potential mistrust and related resistance from community members, some CHWs could be tempted to manipulate data entry on their phones to be able to meet the reporting goals.

#### CHWs short-term commitment and involvement

Respondents highlighted that most CHWs in urban areas often relocate to different places after training, making tracing them quite difficult. More still, there is a decreasing priority of CHW work as it is less facilitated besides being voluntary when they get more responsibilities in their homes or possibly get better job opportunities.

### Benefits that may be realised from mHealth for CHWs for iCCM data

Many benefits stand to be achieved if the above barriers are effectively addressed.

### Institutional and policy benefits

Six subthemes revealed how institutional and policy benefits may be realised through mHealth among CHWs in Banda informal settlement. These were timely data submission, improved data quality, geo-mapping and enabled data authentication, improved CHW monitoring and evaluation, enhanced Community health surveillance and referral, and reduced transport costs for CHWs.

#### Timely data submission

mHealth had the potential benefit of improved data timeliness. According to the KI, with smartphones, CHWs could quickly and efficiently submit data from the field, reducing delays and ensuring more up-to-date information on the health status of their communities. Digitalised data reporting would enable CHWs to promptly report their data daily and weekly, leading to more efficient data collection and management.

*“mHealth will improve the VHT’s data reporting. This is because it would enhance the daily and weekly reporting of data by the CHWs which would also enable them to report in time.” KI 3*.

*“…. through mHealth, the timeliness of reports by the CHWs in the field will be improved” KI 2*.

#### Improved data quality

Using mHealth was thought of as an opportunity to improve overall data quality. The CHWs pointed out that issues like poor handwriting, misplacement of papers, and spelling errors, especially for CHWs with limited education and access to reference materials, will be minimized. The use of closed-ended questionnaires between patients and CHWs in the mHealth app is expected to contribute to this improvement. Improving data management by enabling systematic arrangement at the community and health facility levels would improve data quality. The ability to record social demographic data separately from other information, such as the type of disease signs and symptoms, would contribute to data organisation at the national level. It would reduce the collection of unnecessary information and improve data uploading to computerised ICCM/HMIS. Using an automated upload system would reduce missing data errors, provide ways for easy tracking, and follow up on data using location and time stamps.

*“The mHealth app will also reduce on the burden of paperwork, for example, poor handwriting, misplacing of the papers, and spelling errors. Spelling errors are mainly because most of the CHWs have little or no education and are not provided with reference materials, where they can at least refer to in case they are faced with such a problem… through mHealth, all these errors will be reduced since it will require a closed-ended questionnaire between the patient and the VHT.” FGD 1*.

*“During the VHT’s submissions, the mHealth app creates an indication for time, both the time of submission and the time the VHT worked on the patient… which will not only improve on the VHT programs in the community but also on the quality of data submitted to the national level.” FGD 1*.

#### Geo-mapping and enabled data authentication

Respondents highlighted the potential benefits of digital features such as Geo-positioning system (GPS) applications in community health. Using GPS systems in mHealth would enable the mapping of different areas and communities from which specific data is collected. This would also enable data authentication. This would further provide an opportunity to localise the reported health conditions within communities, improving response and planning for community health.

*“using the GPS coordinates will not only help to give authenticity to the data reported in the field but also enable mapping of different areas in which the data is collected. This will further provide an opportunity to localize the reported conditions to specific communities of attachments by the CHWs” KI 2*.

*‘Smartphones will involve tracking the work activities done by the CHWs in their communities of attachments and through mapping the different coordinates in different communities of attachments, they will be able to find out those CHWs who are actively providing the services to the community members and those ones who are literally doing nothing within the community.” KI 8*.

#### Improved CHW performance monitoring, evaluation, and accountability

mHealth may enhance CHW performance monitoring, evaluation, and accountability by making it easier for supervisors to track activities and performance of different CHWs without necessarily physically going to communities. It would contribute to improved evaluation and accountability of CHWs’ performance and related planning at the community level. It would enable CHWs to monitor and evaluate their performance and associated accountability of health supplies at the community level.

*“The mHealth system would track the VHT’s workload or what they are doing in terms of VHT support… instead of the VHT supervisor going back to the communities of attachments of CHWs time and again to track their activities, which will also reduce on the time the VHT supervisors spend following up activities in the community which will also make supervising much easier.” KI 1*.

*‘mHealth would be very helpful in building accountability or value for money because the system will electronically summarise the total number of households in different communities from whom data has been collected and therefore planning for these households by the Ministry of Health will be made.” FGD 1*.

#### Enhanced community health surveillance, response and referral

CHWs perceived mHealth as an opportunity that would enable them to effectively contribute to community health surveillance activities and enhance the referral system and the necessary quick responses. For example, they said that performing their work with smartphones would enable them to take photos/videos of some rare diseases seen in communities—to build interest amongst health workers to investigate the disease and quickly find solutions.

*“….as we were moving around the community, …, we met a child with some disease that we did not understand, but because we had smartphones, we were able to record this child and sent a video to our in-charge who then sent some health workers to come to that particular community such that they can help this child. I think, through mHealth, we will work even better.” FGD 1*.

“*The mHealth app will not only improve on data quality but also on evaluation of the health activities it creates a summary of the data collected at different time periods. Therefore, before we submit our data collected from communities, each VHT will get an idea about the health problems, the number of people suffering from a particular disease in his/her area which will create evidence in case they supply a few drugs in their health facilities’ FGD 1*.

*“For the patients who leave their referral forms at their homes, the health workers will not record down the references in the hospital records, so CHWs who have referred the patients will not have any records at the healthcare facility, which makes it difficult to assess the referrals for different CHWs. However, if these organisations had some electronic mechanisms…this will be very helpful’ FGD 1*.

#### Cost-effective data reporting

Key Informants reported that using digitalised data reporting through mHealth would be more cost-effective than relying on manual data collection tools. This means that the resources needed for manual data collection, such as paper-based forms and travel costs, can be reduced.

“*……. will save time, money and transport that we use to travel to our health care facilities of attachments to get information about the health status of our communities, of which there are challenges at our health facilities…” FGD 1*.

*“…it would be cheaper if all the CHWs are able to report the data in a digitalised way, other than using data collection tools manually which are sometimes not available.” KI 3*.

### Individual benefits

The subtheme of empowering CHWs with technology revealed the theme of individual benefits of mHealth among CHWs.

#### Empowering CHWs with technology

CHWs perceived mHealth as a way to empower CHWs and a form of upgrade in their type of work. They saw it as an opportunity to ease community health work by highlighting how mobile phones are portable and easier to use compared to difficulties in handling paper copies in communities. CHWs were so excited because it widened their opportunities, including using Google for the first time, which was exciting and empowering to collect good-quality data. Key informants said that working with mHealth motivated them and improved their abilities.

*“mHealth will transform us from using analogue materials to digital…” FGD 1*.

*“It has impacted a lot of efficiency, knowledge and insights towards data quality. Therefore, in my own view, everything is possible provided these CHWs are empowered and have a positive attitude towards technology, there will be a high level of transformation” KI 6*.

## Discussion

This study sought to determine the barriers and benefits of mHealth among CHWs in Banda parish, Kampala. The results of this study reveal institutional and policy, interpersonal and community barriers, and individual barriers and benefits that may affect the implementation of mHealth for CHWs involved in iCCM data collection in the Banda informal settlement. These barriers and benefits can significantly impact the usability, acceptability, and sustainability of mHealth among CHWs.

Limited funding may come with several barriers for mHealth. Rigorous training of CHWs is resource-intensive, creating a high cost [24]. Travel, trainers, and classroom costs contribute to the highest start-up and incremental costs [24] during traditional didactic training. However, using a blended eLearning approach may result in cost-effective and sustainable training [24]. Smartphones are expensive and may take a third of the start-up capital while developing the software, modules, and piloting contribute to over a third of this cost [25]. In addition, smartphones are associated with monthly data plan charges, increasing the cost of implementation. Purchasing cheaper phones may address the high initial start-up cost; however, compromising quality over cost may create technical challenges, such as malfunctioning charging, screen freezing or blank screens, unresponsive keypads or touch pads and malfunctioning SIM cards [26]. Initial start-up costs may be offset by using feature phones, and recurrent data costs can be minimised by using SMS and voice communication and cost-sharing with partners. Inadequate financial and non-financial incentives for CHWs contribute to the existing demotivation, which may also affect mHealth programs [27, 28]. The absence of supplies such as job aids, medicine, and rapid diagnostic tests necessary for implementing their work demotivates CHWs [29, 30]. Insufficient transport facilitation also limits CHWs’ work [31]. Therefore, it is essential to provide regular supplies and transport facilitation to enhance and motivate CHWs’ overall performance [30].

Unreliable power supply and costly internet connectivity disrupt data collection and transmission, impacting program efficiency. Poor mobile network coverage affects usability and has been reported to result in frustration such as delays in meetings, missing participants, and failure to contact emergency services [32]. It also results in data submission delays, affecting data timeliness. Intermittent power supply and phones with low battery life may limit phone usage [33, 34], affecting mHealth intervention implementation. Although households in this study area had access to electricity, some households did not have direct access, and households spend an average of USD$ 10 a month on electricity, which may be unaffordable for community members given their low socioeconomic status [35]. The purchase of a spare battery or power bank may counter this, as reported in Ghana [32]. A portable solar power source may also be useful in recharging phones. Disabling applications that have considerable data usage may also save phone battery.

Phone misuse and theft may result in data loss risks, mostly at home and in public transport, especially in areas with a high crime rate [26]. This study found that working late into the evening and the high crime rate in this community contribute to mobile phone loss and theft. It may also involve CHW assaults and data breaches. Promoting phone safety as part of a robust security measure should be a collective effort, and using feature phones manufactured for CHW tasks with no other functionalities may reduce theft risks.

Community mistrust in mHealth programs may arise from the belief that CHWs may breach privacy and confidentiality [36] and a concern that partners may access this private information [37]. In addition, potential political interference due to a lack of approval and high-level stewardship may hinder mHealth interventions [38]. To boost community trust, programmes should ensure strong community-based engagement right from the selection of the CHWs, choosing CHWs with pre-existing relationships with community members [36], and CHWs who possess linguistic and cultural diversity of the population they serve [31, 37]. Creating awareness of mHealth for CHW through community sensitisation involving community leaders and healthcare providers using multiple communication channels is also crucial in building community trust. Necessary precautions should be taken to ensure the data security of the participants’ information during the data storage and transmission. Legal protection and malpractice should be addressed before program roll-out. Install passwords for applications before using safeguard information on shared devices or in the event of loss or theft. Encryption or scrambling information that only the sender and receiver can decode and decipher may protect data during transmission [34].

Low skills and literacy levels among CHWs, especially older ones, hinder their adaptability to mHealth technology. The design of the question formats involving multiple tasks such as checking boxes, highlighting answers, scrolling horizontally or vertically, free text entry, and touchscreen contribute to the initial difficulty [39]. Low literacy levels create difficulty in reading instructions and understanding the design and workflow of the applications [36, 40]. This is even harder for older users as they lack familiarity with technology. Age has been reported to be negatively associated with using and adopting mHealth technologies among CHWs [40]. Most of these older CHWs have been serving their communities for a long time and are good at community mobilisation and healthcare services. The learning curve for these individuals might be steep, and embracing mHealth may be time-consuming. Simplifying question formats by reducing tasks can make mHealth easier to use. Providing clear instructions, guidance, and support and building confidence can help older CHWs overcome this barrier [41]. Assigning technological roles to younger, tech-savvy CHWs while older ones focus on community mobilisation may be a practical approach, while training and voiceovers can aid in addressing literacy issues.

Data manipulation, which may take the forms of over-reporting progress, underreporting indicators, completely hiding data indicating low progress, and retrofitting service data to match inventory data, affects data quality. Data manipulation may arise from (i) CHWs having limited discretion over the implementation of the activities whose data they collect and yet there is no demonstrated demand for accurate data from their supervisors (ii) Pressure to manipulate data in a bid to exaggerate progress, and (iii) CHWs rationalizing data manipulation to secure their jobs, for personal financial gain, and to cope with pressure from supervisors if present [42]. Data manipulation directly affects data quality, undermining one of the significant aims of mHealth interventions. Data integrity measures, as well as oversight and supervision mechanisms, can prevent data manipulation.

Trained mHealth CHWs’ relocation or prioritising of other responsibilities, resulting in short-term commitment and involvement, disrupts the continuity of healthcare services and community relationships, impacting healthcare delivery effectiveness. There is a need for strategies to enhance CHW retention and long-term engagement in mHealth programs.

Despite the barriers, the study registered significant benefits that could be realised from mHealth among CHWs for iCCM. mHealth improves the timeliness of data collection and submission. With smartphones, CHWs can quickly and efficiently submit data from the field, reducing delays and ensuring more up-to-date health information for their communities. It allows for real-time data transmission, which is crucial for effective decision-making and response [43]. Real-time data collection through mHealth also contributes to improved data quality. Reducing delays and eliminating manual data entry errors associated with paper-based systems enhance the accuracy and reliability of health data [44, 45]. Closed-ended questionnaires within the mHealth app contribute to this improvement, streamlining data collection and management. Timely data submission through mHealth empowers healthcare providers and policymakers with up-to-date information that facilitates evidence-based decision-making, essential for effective resource allocation and response planning [46].

Digital features, including GPS abilities, enable the mapping of different areas and communities and enhance data authentication, and it is a well-established practice in public health [47]. GIS tools allow for the spatial analysis of health data, which is crucial for understanding disease patterns, identifying high-risk areas, and planning targeted interventions. GPS-enabled mHealth applications enhance data authentication by providing precise location data [48]. Accurate geographic data aids in resource allocation decisions. It allows health authorities to allocate resources such as healthcare personnel, medical supplies, and infrastructure where they are most needed [49]. This targeted allocation enhances the efficiency of healthcare delivery. This approach improves data accuracy, aids in response planning, and enhances resource allocation in healthcare. Pinpointing health issues at the community level is particularly crucial for effective public health interventions. These findings underscore the potential of technology-driven solutions to address health disparities and improve the overall well-being of communities.

Monitoring and evaluating CHWs’ performance is more efficient and cost-effective through mHealth. Supervisors can track activities and performance in real-time without physically visiting communities, leading to better evaluation and accountability [50]. mHealth streamlines the monitoring and evaluation process of performance and health supply planning [51]. mHealth streamlines the monitoring and evaluation, leading to more efficient performance management. Moreover, it enhances accountability and informs resource planning and allocation. Real-time data collection and submission profoundly impact community health surveillance and referral systems [52]. Smartphones to document rare diseases enable CHWs to share photos or videos with healthcare workers for quick response and investigation, aligning with the growing emphasis on community-based surveillance. It enables CHWs to report disease outbreaks promptly, track health trends, and initiate rapid response measures, which is crucial for preventing the spread of diseases [53]. Digitalised data collection and reporting through mHealth is more cost-effective than manual data collection. By reducing the need for manual data collection tools and physical transportation of paper forms and data, mobile technology saves resources and time, making healthcare programs more efficient and sustainable [54, 55]. In addition, mobile phones are more portable and user-friendly than paper-based tools. This upgrade simplifies CHWs’ work methods, thereby empowering them. This empowerment motivates CHWs, improves their abilities, and enhances work efficiency [56]. This empowerment is crucial for sustaining CHW programs and improving healthcare delivery in underserved areas.

This study registers strengths as it comprehensively assesses various aspects of mHealth implementation among Community Health Workers (CHWs), including institutional, policy, interpersonal, community, and individual factors, providing a holistic view of the challenges and benefits. It offers practical recommendations for addressing the identified barriers, which can be valuable for policymakers and implementers looking to improve mHealth programs for CHWs. It recognises the potential benefits of mHealth in improving healthcare services for vulnerable populations, emphasizing its relevance in low-resource settings.

However, this study also has limitations that include (1) the study is conducted in a specific geographic area (Banda parish, Kampala), which may limit the generalisability of its findings to other settings, (2) while the study highlights various barriers and benefits, it lacks quantitative data to provide statistical significance, making it challenging to determine the magnitude of the reported effects, (3) the study found more male CHWs were engaged, possibly due to income incentives, but it does not explore this aspect in detail or assess potential gender bias in CHW selection, and (4) the study conducted only two FGDs, however, the follow-up 12 KIIs were able to elaborate on and saturate on the findings from the FGDs. Future studies may explore deeper certain findings, such as gender-related factors.

## Conclusion

This study sheds light on the critical role of mHealth in addressing challenges CHWs face while also recognising the considerable benefits it can bring to healthcare systems, particularly in low-resource settings. Despite barriers such as limited funding and capacity, adopting mHealth technology with appropriate contextualisation regarding the available and possible resources can revolutionise CHW programs and improve the quality of care delivery, offering a promising path toward strengthening CHW programs and enhancing healthcare services for vulnerable populations.

### Electronic supplementary material

Below is the link to the electronic supplementary material.

Supplementary Material 1

## Data Availability

The datasets used and analysed during the current study are available from the corresponding author on reasonable request.

## Abbreviations

CHW: Community health worker
FGD: Focus group discussion
HCF: Healthcare Facility
HMIS: Health Management Information system
ICCM: Integrated Community Case Management
KCCA: Kampala Capital Council Authority
KII: Key informant interview
LMICs: Low middle-income countries
mHealth: Mobile health
MOH: Ministry of Health
MUAC: Mid-upper arm circumference
NGOs: Non-governmental organization
RA: Research Assistant
VHT: Village Health Team
VHT/iCCM: Register Village Health Team register for Integrated Community Case Management register
