# Supplementary material for: Barriers and benefits of mHealth for community health workers in integrated community case management of childhood diseases in Banda Parish, Kampala, Uganda: a cross-sectional study
Source: BMC Prim Care. 2024 May 20;25:173. doi: 10.1186/s12875-024-02430-4 (PMC11103880; doi:10.1186/s12875-024-02430-4)
Supplement: Supplementary file 1 — Supplementary Material 1 [file 12875_2024_2430_MOESM1_ESM.docx]

# Annex

## Annex 2. Key informant Interview Guide for Ministry of Health, KCCA, Partner organisations and Healthcare facility officials

**FOR OFFICIAL USE ONLY**

Date of interview: ______/ _______/ ____________

Name of organisation: _______________________________

Venue: ____________________

Language of interview: _______________________

Time interview started: ________________ Time interview ended: _________________

Key Informant Interview identifier: _____________________________________

**Socio demographic characteristics of the participants**

| **Date of interview** | **Response** |
| --- | --- |
| **Sex** |  |
| **Profession** |  |
| **Position** |  |
| **For how long have you been in that position?** |  |
| **Level of education** | 1. **No formal education** 2. **Primary** 3. **Secondary** 4. **Tertiary** |

**Guiding questions: based on Topic guides by the M-RDQA tool (MEASURE, 2007)**

**Quality of data collected by VHTs**

*HMIS Capabilities, Roles and Responsibilities (Data collection for the VHTs)*

1. Who is responsible for collection and consumption of HMIS-ICCM data from the village level up-to the national level?
2. Who is responsible for consumption of HMIS-ICCM data from the village level up-to the national level?

*Training of VHTs for data collection*

1. How are the VHTs trained upon recruitment? (Probe on data collection, VHT register as a tool for data collection, VHT/ICCM register as a tool for data collection?)
2. How often are VHT retrained? (Probe for: refresher courses, capacity building mechanisms/session)

*Indicator Definitions*

1. How are the technical/medical terms including different diseases and equipment used in HMIS-ICCM defined/described/explained to VHTs? (Probe: during training.
2. Are VHTs provided with reference materials for these definitions? If yes/no, elaborate on the kind of ref materials.

*Data Reporting Requirements*

1. What do VHTs require in order to report accurate timely data from their villages? (Materials, facilitation etc.)

*Data Collection and Reporting Forms and Tools*

1. What tools are given to VHT for collection and reporting of Data? (Probe: VHTs registers, VHT/ICCM registers and summary forms for VHT supervisors?).

*Data Management Processes and Data Quality Controls*

1. How is the quality of data from VHTs managed/ensured at different levels?
2. What are the feedback and supervision processes for VHTs? (Probe: Who supervises/oversees quality of data collected by VHTs, how often/when etc, what are the requirements/what is necessary for effective supervision)?
3. How are supervisors trained on their duties? (i.e. on checking data:
4. accuracy–reliability (data measure what they are intended to measure and measures do not change according to who is using them and when or how often they are used)
5. completeness
6. timeliness (up-to-date and available on time)
7. availability (availability and accessibility of the records when needed)
8. Integrity/spot check (no deliberate bias or manipulation).
9. How is their training different trainings from the other VHTs?
10. What happens when poor quality data is found/reported? What are the mechanisms of dealing with poor quality data?
11. What do you think are the a) enablers, and B) barriers to of VHT data quality?

*Links with National Reporting System*

1. How is data collected by VHTs linked to the national HMIS data?

**Barriers and opportunities for mHealth**

1. What are your perceptions on using mHealth by VHTs for ICCM?
2. Do you have any experience of projects involving/incorporating mobile phones into healthcare? If yes, please elaborate.
3. In what ways do you think mHealth could improve VHT programs including ICCM data collection? Probe: What are the opportunities/benefits of integrating Mhealth into ICCM activities?
4. What do you think are the potential challenges of mHealth integration into ICCM and VHT programs?

## Annex 3. Focused group discussion guide

**Guiding questions**

1. What are your perceptions on using mHealth by VHTs for ICCM?
2. Do you have any experience of projects involving/incorporating mobile phones into healthcare? If yes, please elaborate.
3. In what ways do you think mHealth could improve VHT programs including ICCM data collection? Probe: What are the opportunities/benefits of integrating Mhealth into ICCM activities?
4. What do you think are the potential challenges of mHealth integration into ICCM and VHT programs?
